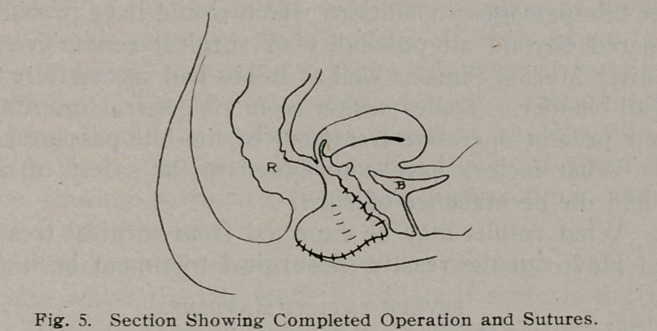# The Treatment of Prolapsus Uteri, and with Special Reference to the Author’s Operation for Ruptured Perineum1Read before the Buffalo Academy of Medicine, Section on Obstetrics and Gynecology, October 26, 1909.

**Published:** 1910-01

**Authors:** Herman E. Hayd

**Affiliations:** Buffalo, N. Y.; Surgeon to the German and the German Deaconess’s Hospitals


### BUFFALO MEDICAL JOURNAL.

VOL. LXV. JANUARY, 1910. No. 6

### ORIGINAL COMMUNICATIONS.

### The Treatment of Prolapsus Uteri, and with Special Reference to the Author's Operation for Ruptured Perineum[1]

BY HERMAN E. HAYD, M. D., M. R. C. S., (Eng.) Buffalo, N. Y.
Surgeon to the German and the German Deaconess's Hospitals

PROLAPSUS uteri shows itself in different degrees of severity, and has been arbitrarily divided into different classes, according to the degree of decensus. It is obvious, however, to all of us, that the condition—no matter what degree—is the result of the same pathological processes, and its treatment must be accomplished by the same measures; and these only differ in degree from simple plastic surgery of the vagina and perineum, with or without vaginal hysterectomy or hysterorrhaphy, to the most difficult, complex and elaborate surgery for the cure of pelvic hernia.

It is usually slow in its development, and, as a rule, is the result of a previously ruptured and unrepaired perineum, together with such relaxation of the soft parts as would be consequent upon a breach in the pelvic floor, and increased by frequent childbearing, lifting of heavy weights, straining at stool, or any other form of intraäbdominal pressure—particularly when the direction of this force is upon the upper and anterior surface of the uterus, as is the case with a retroposed uterus, so often the antecedent of prolapsus uteri. It is occasionally acute in its onset, and it is occasionally seen even in the nulliparous woman. when it is the result of some severe fall or violence which pushes the uterine body down into and through the introitus vaginæ. It may be accompanied by painful and perhaps very dangerous and distressing symptoms, due to the traumatism; but, just as soon as the acute trouble subsides the subsequent history and treatment would be similar to the more gradually developed cases.

The various methods of treatment which are employed must depend upon the degree of prolapsus, the age and social con-

---

1. Read before the Buffalo Academy of Medicine, Section on Obstetrics and Gynecology, October 26, 1909.

dition of the patient, whether the woman has borne children, and perhaps has a large family and is the wage-earner of her family. Tentative measures, such as tampons, balls and pessaries, can accomplish but little in the way of permanent relief with this distressing condition, and, therefore, surgical interference sooner or later becomes necessary and imperative. However, women of means, who are removed from the necessities of work and arduous labor can often be made very comfortable by carefully applied local measures.

Minor degrees of prolapse can often be cured by bringing the retrodisplaced uterus forward by an Alexander operation, and then performing an anterior and posterior colporrhaphy and perineorrhaphy. These operations hold the uterus forward and upward and give the broad ligaments a chance to retract. The round ligaments have but little power to lift the uterus upward—they simply pull it forward, while the plastic operations narrow the vaginal canal and further support and elevate the uterus.

When the body of the uterus presents at the introitus, or the cervix comes into the world, hysterectomy—vaginal or abdominal—with anterior and posterior colporrhaphy and perineorrhaphy, or a ventral fixation with the same plastic surgery of the vagina are indicated, and in most cases they prove permanently successful. In a woman beyond the child-bearing period in whom the necessities and promptings for children does not exist and where the rightful demands of the state and society cannot be satisfied, I believe it is the best practice to remove the uterus in cases of bad prolapsus because the result usually is much more satisfactory. If, however, the woman be young and has a healthy functionating organ with good tubes and ovaries, the uterus should not be sacrificed, even though the cervix be badly torn and eroded, because a ventral fixation combined with efficient vaginal plastic surgery is generally curative. Amputation of the cervix is the operation of choice when the cervix is lacerated or is long and pointed, because we then remove the wedge which dilates and pushes downward the soft parts and which is such a potential factor in the production of the increasing decensus.

There are many objections to fastening the uterus on to the abdominal wall. The patients often suffer from frequent hemorrhages, pain and tenderness in the scar and lower abdominal region, irritable bladder, and the like.

Then, too, the cervix and soft parts often tear loose from the fixed uterus above and permit a return of the cystocele and rectocele, and even a prolapse of the cervix through the introitus and yet the body of the uterus will remain firmly fastened to the abdominal wall, due to a supra vaginal elongation of the cervix, which permits and encourages the descent of the soft parts below. This occurred in three of my operated cases; in two I subsequently removed the uterus per vaginam, and then again took up the cystocele and rectocele and sewed the vault of the vagina to the stumps of the broad ligament with a perfect and lasting success.

The other case fell into the hands of Dr. Howard Kelly, who removed the uterus per vaginam and closed up the vagina intentionally so that future intercourse was impossible. The result, so far as the prolapsus is concerned, is most satisfactory. However, any procedure which robs a woman of her connubial happiness is not often necessary, nor will she tolerate such surgery, and therefore it should be undertaken only after every other means had failed, because it brings with it so much domestic unhappiness; not only is the husband compelled to lead a life of sexual abstinence, but the wife, by being the cause of this forced restraint, broods over her condition and becomes depressed, hypochondriacal, sometimes even suicidal.

Therefore, as I have previously stated, I prefer to remove the uterus in the treatment of procidentia uteri, and sometimes do it per vaginam or per abdominem, depending in a measure upon the degree of flaccidity of the broad ligaments and the extent of the pelvic hernia. There are several advantages in doing an abdominal hysterectomy, the vault of the vagina and the broad ligaments and round ligaments can be better sewed together in one large firm stump, but this stump will again fall, if the pelvic outlet has not been narrowed by good plastic surgery. I usually complete all the operations at one sitting; but, if for any reason, it is found impossible, then I do the abdominal work later.

Occasionally the bladder and rectum will prolapse after the uterus has been removed, but I am satisfied this is more often the result of poor plastic surgery on the anterior and posterior vaginal walls. The split edges of the obturator or pelvic fascia with the levator ani muscle were not carefully fished out and brought together and retained in apposition by a special layer of chromic gut sutures, and unless this is carefully done failure is inevitable.

I have had a number of disappointments, where I have fixated the uterus on to the abdominal wall, and with the various round ligament operations, due to the too early absorption of the chromic gut or kangaroo tendon which I had employed; consequently, I have given up the use of all absorbable ligatures in clean surgical cases, when I want a long and firm attachment between tissues and invariably sew these structures together with Pagenstecher's celluloidzwirn or linen thread. Certain peritoneums digest and absorb these materials so quickly that one cannot depend upon them, since one cannot foresee in whom this solution will take place prematurely. I never use silk or linen thread in pus cases.

Sometimes the procidentia is extreme, so that the uterus and vagina fall down between the legs, even to the knees, and the contents of the sac consist not only of rectum and bladder, but also of coils of the small intestine. In one such extreme case upon which I operated successfully five years ago, I sewed the narrowed vagina and broad ligament stumps into one firm piece and then quickly did an abdominal section and sewed the stump with kangaroo tendon to the abdominal wall, and the soft parts, bladder and rectum have remained in splendid position ever since. Many elaborate operations have been devised to cure this condition of pelvic hernia, and one but recently described by Dr. Polk of New York, but all have as their basis a very free dissection of the vaginal structures, narrowing of the vagina and in-

Fig. 1. Diagram of Posterior Vaginal Wall Showing Denuded Area.

troitus and a reduplication or taking up of the overstretched and thinned-out broad ligaments, and all require as a foundation, to prevent a recurrence, a strong, firm and unyielding perineum.

Operations for cystocele, whether associated with a prolapsed uterus or not, are not so uniformly successful in my hands as the operation of posterior colporrhaphy and perineorrhaphy when done for a rectocele, and I am sure it is because in many cases the anterior fascia is not torn in the median line, but laterally along the rami of the pubes and ischium, and therefore cannot be found through the ordinary diamond-shaped cystocele denudation or by the Stolz operation, and the fascia is, therefore, not picked up and brought together as it can be in the perineal operation with the finger in the rectum to differentiate the fascia and levator ani muscle. Fortunately, a slight giving away of the anterior wall is not so serious if the perineum and posterior vaginal wall be permanently fixed, because the cystocele will not recur to any degree, as the fixed perineum will prevent its descent.

I wish now to describe my operation for rectocele and ruptured perineum, which consists of a free posterior colporrhaphy and perineorrhaphy and the taking up of the pelvic fascia and levator ani muscle, where they are torn, and the sewing up of the various structures from above downward in the direction that these tears take during parturition.

We have here a section of the posterior wall of the relaxed and torn vagina with the cervix on top. A curved, sharp-pointed scissors is pushed into the junction of the skin of the perineum and the mucous membrane of the vagina, in front of the anus. The mucous membrane of the vagina is then freely separated from the skin and other structures, as far as the carunculæ or those points—one on each side—to which the perineum will be closed. Then this piece of separated mucous membrane is picked up with a pair of artery forceps and divided across with a scissors. A double incision is now made with angular scissors up

Fig. 2. Buried Sutures for Levator Ani and Obturator Fascia.

Fig. 3. Upper Part of Wound Brought into Position and Sutured.

the posterior central portion of the vaginal wall even to the cervix uteri, depending upon the amount of relaxation and flaccidity of the vagina, and a piece of mucous membrane, triangular in shape, is removed, from a half to one inch in width, according to the size of the vagina (Fig. 1). The finger is then passed high up into the rectum and pressure is made forward, and wherever there is a break in the pelvic floor the hard edges of the obturator fascia and levator ani muscle will be felt, when they are carefully brought together and sewed with fine chromacised catgut, and always tied by the assistant, so as to avoid infection and then when the finger is removed, it is washed and a cot is put on and the subsequent stages of the operation are completed.

This sewing process is commenced high up in the vagina and is continued downward until the whole fascia and levator ani muscles are perfectly approximated. The bulging rectum is turned in on itself and its lumen is lessened by taking up the excess with a few stitches of catgut. Then the vaginal mucous membrane is brought together by interrupted sutures to the introitus and the perineal edges of the wound with silkwormgut: the most anterior suture also takes in, not only the skin, but the lowest part of the opposed vaginal sides, which we may call the crown stitch. It will now be seen that we have lengthened the vagina and changed its horizontal course to an oblique one, and have made a strong, full perineal body, which any effort at coughing or straining will not displace. Moreover, in some cases where the perineum is very much relaxed and stretched out, not so much from the tearing of the fascia and levator ani muscle as from simply the stretching of them. Here through this high and ample denudation which is provided in my operation, the loose fascia and muscle can be picked up and lapped on itself, and in this way

Fig. 4. Skin Sutures to Close Lower part of Wound and Perineum.

the relaxed structures may be permanently fixed by the buried chromic gut sutures.

Previous to this operation I did the Emmet and was taught the operation by Dr. Mann, whom I assume does the classical operation as well as anybody else, since he was one of Emmet's pupils and house surgeons. I had so many failures with the operation as I did it, that I began doing this one, which I have gradually elaborated, and which has proved very successful in my hands, and with it I have cured other cases on which I failed by a previous Emmet or bat-wing denudation.

Its advantages are:

1. It brings up the fascia and levator ani muscle into view, and their closure can be perfectly accomplished.

2. It removes the central and most stretched-out portion of the vagina, and therefore the most distensile part, and exposes by reason of the denudation the dilated and distended rectum, into which a few stitches of catgut can be placed, and thus tuck up or lap up the excess of rectal pouch.

3. It lengthens the vagina by converting a horizontal into an oblique canal.

4. It provides a thick permanent perineal body instead of a skin perineum, which so often results from other operations.

5. It brings up the several layers of tissue and coapts them in the direction in which the tear usually took place, and secures

Fig. 5. Section Showing Completed Operation and Sutures.

in perfect apposition adjacent and similar plains of structure and holds them, muscle to muscle and fascia to fascia by buried absorbable sutures, until firm and perfect union results.

493 Delaware Ave.

## Figures and Tables

**Fig. 1. f1:**
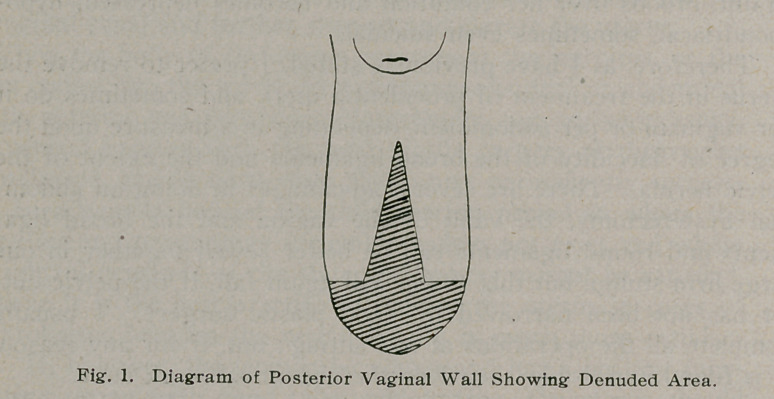


**Fig. 2. f2:**
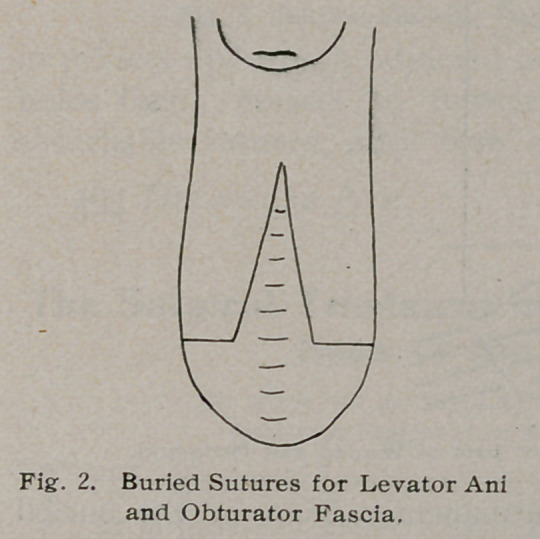


**Fig. 3. f3:**
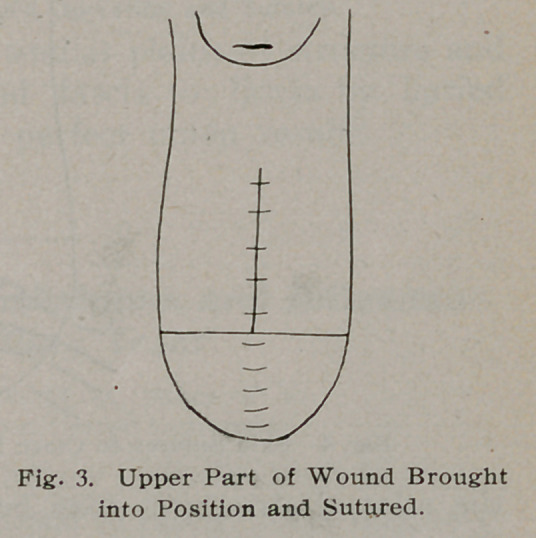


**Fig. 4. f4:**
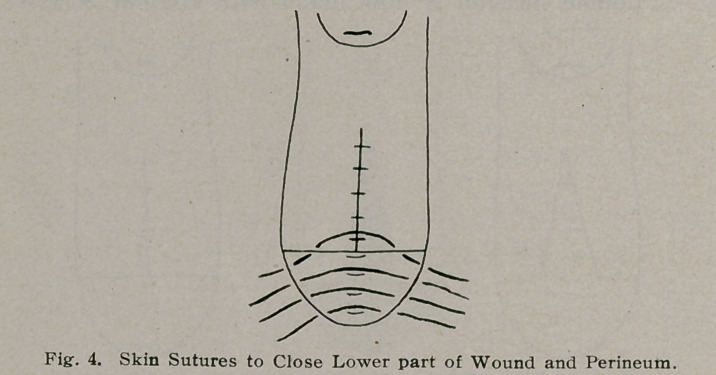


**Fig. 5. f5:**